# Genome-Wide Association Study Identifies That the ABO Blood Group System Influences Interleukin-10 Levels and the Risk of Clinical Events in Patients with Acute Coronary Syndrome

**DOI:** 10.1371/journal.pone.0142518

**Published:** 2015-11-24

**Authors:** Åsa Johansson, Jenny Alfredsson, Niclas Eriksson, Lars Wallentin, Agneta Siegbahn

**Affiliations:** 1 Department of Immunology, Genetics and Pathology, Science for Life Laboratory, Uppsala University, Uppsala, Sweden; 2 Uppsala Clinical Research Center, Uppsala University, Uppsala, Sweden; 3 Department of Medical Sciences, Clinical Chemistry, Uppsala University, Uppsala, Sweden; 4 Science for Life Laboratory, Uppsala University, Uppsala, Sweden; Roswell Park Cancer Institute, UNITED STATES

## Abstract

**Introduction:**

Acute coronary syndrome (ACS) is a major cause of mortality worldwide. We have previously shown that increased interleukin-10 (IL-10) levels are associated with poor outcome in ACS patients.

**Method:**

We performed a genome-wide association study in 2864 ACS patients and 408 healthy controls, to identify genetic variants associated with IL-10 levels. Then haplotype analyses of the identified loci were done and comparisons to levels of IL-10 and other known ACS related biomarkers.

**Results:**

Genetic variants at the ABO blood group locus associated with IL-10 levels (top SNP: rs676457, *P* = 4.4 × 10^−10^) were identified in the ACS patients. Haplotype analysis, using SNPs tagging the four main ABO antigens (A1, A2, B and O), showed that O and A2 homozygous individuals, or O/A2 heterozygotes have much higher levels of IL-10 compared to individuals with other antigen combinations. In the ACS patients, associations between ABO antigens and von Willebrand factor (VWF, *P* = 9.2 × 10^−13^), and soluble tissue factor (sTF, *P* = 8.6 × 10^−4^) were also found. In the healthy control cohort, the associations with VWF and sTF were similar to those in ACS patients (*P* = 1.2 × 10^−15^ and *P* = 1.0 × 10^−5^ respectively), but the healthy cohort showed no association with IL-10 levels (*P*>0.05). In the ACS patients, the O antigen was also associated with an increased risk of cardiovascular death, all causes of death, and recurrent myocardial infarction (odds ratio [OR] = 1.24–1.29, *P* = 0.029–0.00067).

**Conclusion:**

Our results suggest that the ABO antigens play important roles, not only for the immunological response in ACS patients, but also for the outcome of the disease.

## Introduction

Acute coronary syndrome (ACS) and coronary artery disease (CAD) are among the major causes of death worldwide [1]. ACS is a clinical manifestation of disrupted atherosclerotic plaques in the coronary arteries. Atherosclerotic plaques consist of mainly inflammatory and immune cells, lipids and debris that are accumulated in the intima of larger arteries. When such plaques are disrupted, inflammatory cytokines are released into the circulation, which leads to inflammation and acute thrombotic events. ACS is represented by a spectrum of clinical symptoms caused by angina and leading to myocardial ischemia. Several factors contribute to an infarction but inflammation plays a key role, not only in initiating, but also for propagating and activating the lesions in the arteries [2]. In ACS patients many of the immune cells have become activated and produce inflammatory cytokines. This leads to elevated levels of circulating inflammatory markers, which in turn reflects the severity of this condition [2].

Furthermore, there is a direct relationship between both the number of circulating and local inflammatory cells and the severity of the coronary syndrome [3,4]. Following a trauma, such as the rupture of an atherosclerotic plaque, both anti-inflammatory and pro-inflammatory cytokines are released into the circulation [5]. Interlukin-6 (IL-6) and Interlukin-18 (IL-18) levels have both generally been regarded as robust risk predictors for cardiovascular mortality due to their pro-inflammatory properties, and they are both strong independent biomarkers for predicting death in CAD patients [3,4]. Interestingly, studies on the anti-inflammatory cytokine Interlukin-10 (IL-10) are contradictory. In some cohorts, elevated IL-10 levels are associated with a favorable prognosis [6], whereas in others, high levels of IL-10 are associated with a poor outcome [7].

In a previous report we found that increased IL-10 levels are associated with a poor outcome in ACS, and that single nucleotide polymorphisms (SNPs) at the IL-10 locus influence the levels of IL-10 in both ACS patients as well as in healthy controls [7]. In the present study we performed a genome-wide association study (GWAS) to further explore the genetic contribution to the IL-10 response in ACS. This was done by genotyping nearly 200,000 SNPs using the CardioMetaboChip genotyping panel (Illumina) in ACS patients and healthy controls. The CardioMetaboChip is specifically enriched for SNPs and gene targets from previously conducted studies on cardiovascular and metabolic diseases. Markers on the CardioMetaboChip were selected based on one of the following two criteria: 1) the markers have been associated with a disease, or a disease related phenotype (e.g. blood pressure changes, insulin resistance, metabolic disorders, dyslipidemia, and inflammation) in a previous GWAS, or 2) the markers are part of a fine mapping panel surrounding a candidate gene for a disease or disease related phenotype.

The aim of this study was to identify genetic loci associated with IL-10 levels, and to refine the results using haplotype analyses. We also studied these SNPs and haplotypes in relation to other known biomarkers of inflammation and coagulation, as well as in relation to the severity and clinical outcomes in the ACS patients.

## Methods

### Study participants

All patients in the ACS cohort were included in the Scandinavian multi-center trial FRagmin and fast revascularization during InStability in Coronary artery disease-II (FRISC-II), registration number: ISRCTN82153174 [8,9]. In total, 3489 patients with ACS were included in the trial and a comprehensive description of the inclusion criteria has been published previously [7]. As part of the clinical trial, study participants were randomly assigned to receive either an early invasive, or a non-invasive treatment strategy, and was given either placebo or long-term low-molecular-mass heparin (dalteparin) for 3 months. Clinical endpoints were followed-up for up to nine years and include: death, cause of death, fatal or non-fatal myocardial infarction (MI). Controls (N = 500) were recruited from the Swedish population registry (SWISCH) [10], and these individuals had no clinical history of cardiovascular disease or cardiovascular risk factors, had a normal electrocardiography and routine blood chemistry. The controls were recruited from all parts of Sweden and were matched pair-wise in terms of age and sex with the same number of patients from the FRISC-II ACS cohort.

### Ethics Statement

Written consent was obtained from all study participants and the study were approved by the regional ethics review board of Uppsala and conform to the principles outlined in the Declaration of Helsinki.

### Sample preparation, biochemical analyses, and clinical endpoints

Blood sampling of the ACS cohort was performed at randomization after admission to the coronary care unit. The median time from admission to blood sampling was 39 hours, with a maximum interval of 190 hours. Venous blood samples from the healthy controls were obtained at outpatients visits. High sensitive IL-10 was measured in all individuals using the Enzyme-linked immunosorbent assay (ELISA) technique with a lower detection limit of 0.5pg/mL (R&D Systems, Minneapolis, MN, USA). Plasma protein levels of von Willebrand factor (VWF), and soluble tissue factor (sTF) was measured in 600 of the FRISC -II patients and all of the controls, while IL-18, IL-6 and CD40 ligand were measured in all FRISC-II patients and all controls using ELISA as described previously [11–13]. Fibrinogen levels were analyzed in all FRISC-II patients and all controls by rate nephelometry using the Beckman array protein system (Beckman Instruments Inc., Paris, France) [14]. The clinical endpoints investigated in this study were: 1) MI within 5 years, cardiovascular, 2) cardiovascular death within 5 years, 3) any cause of death within 9 years, and 4) any cause of death or MI within 5 years. These endpoints have previously been described in detail [15].

### Genotyping

DNA was extracted from patients and controls as previously described [7]. Genotyping was performed according to the manufacturer's instructions for a total of 3606 samples (ACS patients and controls with samples available for DNA analysis) using the Illumina CardioMetaboChip Genotyping BeadChip (Illumina, San Diego, CA). Analysis of the raw data was performed using Illumina GenomeStudio 2009, the Illumina Infinium II assay, and using Illumina’s reference clusters [16]. Genotyping as well as quality control (QC) were performed in ACS patients and controls together.

### SNP quality control

Individuals with duplication errors were removed and an individual call rate of 98% was applied. Samples where autosomal heterozygosis was too high (FDR<0.01), and which failed the X-chromosomal sex check were also removed. After the QC a total of 3345 individuals remained in the study, of which 2864 ACS patients and 408 controls were analyzed for IL-10 levels. A total of 185801 SNPs with an average call rate of 99.58% were extracted from the CardioMetaboChip. Of these, 183282 (98.6%) had a SNP call rate >98%, the quality cut-off recommended by Illumina. To remove all non-polymorphic SNPs a cut-off for minor allele frequency (MAF) of 0.001 was used. All SNPs severely out of Hardy-Weinberg equilibrium (HWE) (*P*<10^−6^) were also removed, and after filtration, 130,332 SNPs remained for further analyses.

### Genetic principal components

ACS patients and controls were sampled from a large demographic region representing Scandinavia. Since the CardioMetaboChip is enriched for target genes that are candidates for being involved in the development of CVD and metabolic phenotypes, it is likely that SNPs with a difference in frequency between the ACS patients and the controls are overrepresented on this chip. To get an unbiased estimate of the principal components, the principal components from the genotype data were calculated using a set of 4559 SNPs that are the least likely to have an influence on CVD phenotypes. These 4559 SNPs were identified in by the CARDIoGRAMplusC4D Consortium [17]. We did not identify any population outliers or clustering of individuals either due to their geographic origin (S1 Fig), or disease status, and therefore we did not use the genetic principal components in our further association analyses.

### Transformation of biomarker trait values

To investigate the association between biomarkers and SNPs, the biomarker values (or residuals) need to be approximately normally distributed and without a heavy tail. Though, most biomarkers are not normally distributed and the values need to be transformed prior to association analyses. Many studies use log transformation of biomarker traits, but in most GWAS, traits are transformed using the rank-bases inverse normal transformation. Rank-transformation generates a standard normal distribution, (mean = 0 and standard deviation [SD] = 1), unless the dataset contains too many ties. This method increases the sensitivity for detecting true positive associations [18]. To avoid false positive results due to non-normality of the residuals, IL-10 values were transformed prior to the association analyses using a rank-based inverse normal transformation, implemented as the rntransform function in the GenABEL R Library [19].

### Statistical analyses

Association analyses were performed for ACS patients and controls separately, and all analyses included sex and age as covariates, while additional precision variables were identified for IL-10 (see results section). For clinical endpoints analyses: history of previous MI, Diabetes, and randomization information were also included. All association analyses were performed using either a linear (for continuous values), or logistic (for clinical endpoints) regression model for genome-wide SNP data, implemented as the mlreg function in the GenABEL R library [19]. The genome-wide threshold for significance was estimated to be *P* = 3.84 × 10^−7^ using the Bonferroni approach to correct for the 130,332 SNPs tested. All *P*-values were adjusted for inflation using genomic control. Haplotypes over the *ABO* region were constructed for each individual using the EM algorithm, implemented in the haplo.em function in the haplo.stats R package. Haplotype score statistics were performed using the haplo.score function [20] in the haplo.stats R library. To test the effect of a trait on each individual haplotype we used a generalized linear regression model for haplotype analyses, implemented in the haplot.glm function [21] in the haplo.stats R library.

### Path analysis

Path analysis is a method for separating direct and indirect effects, and for measuring the relative association of any causal factors involved amongst a set of variables. For the ACS patients we included: sex, age, acetylsalicylic acid (ASA) treatment, statin treatment, IL-10, VWF, ABO antigen, and incidence of death after nine years as variables. For the controls the same variables were included with the exception of death as this information was not available. As the previous analyses of the 0 and A1 antigens showed similar effects on IL-10 and VWF (S2 Table), we clustered all individuals into either: 1) individuals with O/O, A2/A2 and O/A2 antigens, or 2) individuals with all other antigen combinations. All possible combinations of associations between the variables were tested. All non-significant associations were removed step-wise leaving only significant coefficients in the final model (*P*<0.05). Path Analysis was performed using the sem R library.

## Results

### Baseline characteristics

Baseline characteristics of the ACS cohort and the healthy controls, as well as IL-10 levels in relation to clinical endpoints for the whole study have been previously published [7], while the characteristics and clinical endpoints for the subset of the patients and the controls included in this study are shown in Table 1. An association with IL-10 was investigated for a number of confounders and precision variables (Table 2), and all significant variables (sex, age, statin treatment at randomization and history of diabetes) were included in the downstream analyses investigating the associations between SNPs and IL-10 levels. In this genetic sub-study, IL-10 levels were significantly higher in the ACS patients compared to the healthy controls (Table 3). In the patients, the IL-10 levels range from 0 to 105.83 pg/mL, but only 18 individuals had a value higher than 10pg/mL. In addition, a large number of values, (21.3% in the control group and 17.5% in the ACD cases), were below the detection threshold of 0.5pg/mL and were thereby set to 0. Both VWF and sTF levels were also higher in the ACS patients compared to the controls (Table 3). The IL-10 values deviated from normality with a heavy tail (S2 Fig), and were therefore transformed in downstream analyses, whereas sTF and VWF values did not deviate from normality and were left untransformed.

**Table 1:** Characteristics of the study cohort.

	Non-ST ACS patients (N = 2864)		Controls (N = 408)	
	**Median**	**Interquartile range**	**Median**	**Interquartile range**
**Age (years)**	67.16	59.1–74.01	65	59–70
**BMI (kg/m^2^)**	26.28	24.26–28.73	25.4	23.17–27.4
	**N**	**Percentage of total**	**N**	**Percentage of total**
**Males**	2016	70.4	272	66.7
**Current smokers**	980	33.6	146	35.8
**Previous MI**	779	27.2	NA ^a^	
**History of Diabetes mellitus**	357	12.5	NA	
**ST depression at admission**	1331	47.6	NA	
**Cardiovascular death within 5 years**	225	7.9	NA	
**MI within 5 years**	472	18.1	NA	
**Any cause of death 9 within years**	590	20.8	NA	
**Death of MI within 5 years**	652	25.0	NA	

This study cohort include genotyped FRISC-II patients and controls with measured IL-10 levels.

^a^NA–Information not available.

**Table 2:** Variables influencing IL-10 levels.

	Estimate	SE	*P*
**Sex (female = 0, male = 1)**	0.17	0.040	2.3E-05
**Age (year)**	0.011	0.0019	4.0E-08
**Current smoking**	0.0016	0.039	0.97
**Previous MI**	-0.020	0.046	0.67
**ST depression at admission**	0.054	0.037	0.14
**Statin treatment at admission**	-0.12	0.058	0.042
**ASA treatment at admission**	0.040	0.043	0.35
**Diabetes mellitus**	0.18	0.055	0.0010

Multiple linear regression using possible precision and confounding variables as explanatory variables and IL-10 as response variable for the association study. The table shows the regression coefficient (Estimate), the standard error (SE) and the P-value (*P*) for each variable.

**Table 3:** Statistics summary of biomarkers in the ACS patients and controls.

	Patients					Controls					
Variable	N	Min	Max	Median	Mean	N	Min	Max	Median	Mean	*P*
**IL-10 (pg/mL)**	2864	0	105.8	1.08	1.6	408	0.5	104.5	0.77	1.2	3.4 × 10^−19^
**sTF (pg/mL)**	757	28	644	161	178.4	402	0	588	124	139.4	6.4 × 10^−18^
**VWF (pg/mL)**	502	26	312	154	153.7	409	56	276	130	132.7	1.5 × 10^−14^

The table shows the summary statistics for three biomarkers in the ACS patients and controls. For each sub-cohort, the number of individuals with the biomarker measured is included together with the minimum, maximum, median and mean value for each biomarker. The last columns who the P-value for the difference in biomarker levels between ACS patients and controlled, estimated by Kruskal-Wallis rank-sum test.

### GWAS

**Fig 1:**
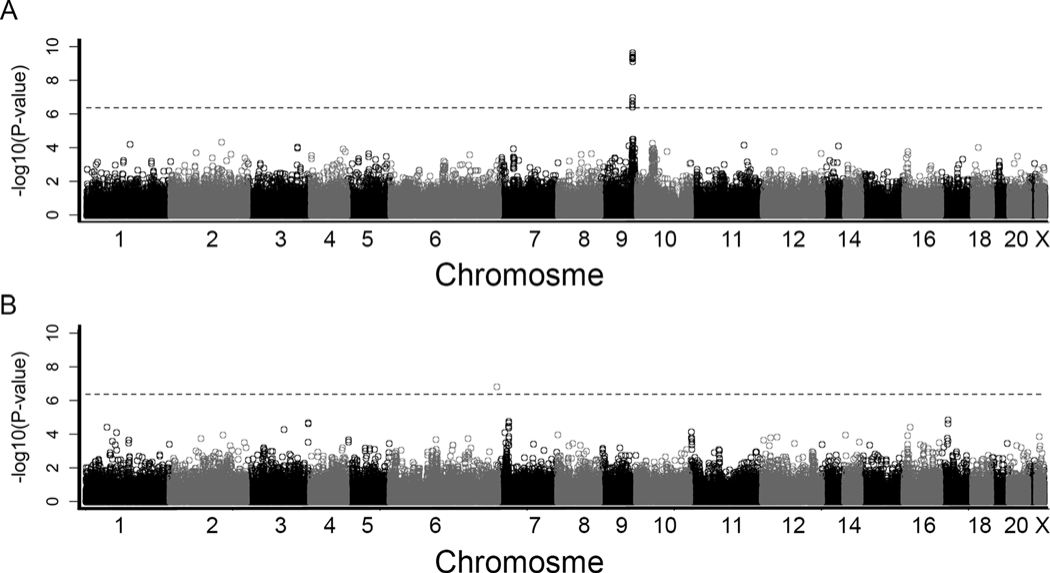
Manhattan plot for the genome-wide association analyses in ACS patients (A) and controls (B). For each SNP, the–log10 (P-value) is plotted against the order on the chromosomes starting with 1–22, Y and X, separated by different colors. The dashed line indicates the threshold for statistical significance (Bonferroni adjusted *P* = 0.05).

Genome wide association analyses of IL-10 levels (S3 Table) resulted in one highly significant peak on chromosome 9 in the ACS patient cohort (top SNP: rs676457, *P* = 4.44*10^−10^), and another borderline significant SNP on chromosome 6 in the healthy controls (rs11961593, *P* = 1.80 × 10^−7^) (Fig 1). The inflation factor lambda was estimated to 1.038 in the ACS patients and 1.019 in the healthy controls (S3 Fig). The association signal in the ACS patients represents a large number of SNPs located within the *ABO* locus on chromosome 9, all with similar allele frequencies and effects (betas). This region consists of three different LD blocks of which one includes the most significant SNPs from our analyses, rs676457 (S4 Fig). The minor allele of this most significant SNP in the *ABO* region (rs676457) is associated with decreased IL-10 levels (Table 4). Increased IL-10 levels are associated with an increased risk of all causes of death and MI (Table 5). This is in agreement the major allele in rs676457 being associated with increased IL-10 levels, as well as poor disease outcome for most of the clinical endpoints investigated in this study (Table 5). Only one SNP (rs11961593) reached the level of genome-wide significance in the control material and is located almost 100kb upstream of the phosphodiesterase 10A (*PDE10A*) gene (Table 4). This variant is not associated with IL-10 levels in the ACS patients, or with any of the clinical outcomes (*P*>0.05), and is most likely a false finding.

**Table 4:** Genome-wide significant SNPs associated with a variation in IL-10 levels.

Cohort	CardioMetaboChipID	SNP name	Chr	Position (Build 38)	Allele	Freq	N	Beta	SE beta	*P*	HWE *P*	Call rate
Patients	chr9:135125967	rs8176702	9	133260743	A	0.376	2857	0.13	0.026	3.82 × 10^−7^	0.87	0.9976
Patients	chr9:135126784	rs2073826	9	133261560	A	0.392	2864	0.13	0.026	4.36 × 10−^7^	0.81	1
Patients	chr9:135126886	rs687621	9	133261662	G	0.381	2863	-0.16	-0.026	7.89 × 10^−10^	0.48	0.9997
Patients	chr9:135126927	rs687289	9	133261703	A	0.381	2864	-0.16	-0.026	1.00 × 10^−9^	0.50	1
Patients	rs2073827	rs2073827	9	133261730	G	0.392	2862	0.13	0.026	5.74 × 10^−7^	0.81	0.9993
Patients	chr9:135129086	rs657152	9	133263862	A	0.398	2864	-0.16	-0.026	9.25 × 10^−10^	0.44	1
Patients	rs8176682	rs8176682	9	133263894	A	0.376	2864	0.13	0.026	2.50 × 10^−7^	0.72	1
Patients	chr9:135129575	rs8176681	9	133264351	G	0.393	2864	0.13	0.026	3.84 × 10^−7^	0.72	1
Patients	chr9:135132024	rs514659	9	133266790	C	0.381	2864	-0.16	-0.026	7.56 × 10^−10^	0.45	1
Patients	chr9:135132038	rs644234	9	133266804	C	0.399	2864	-0.16	-0.026	8.03 × 10^−10^	0.41	1
Patients	chr9:135132176	rs643434	9	133266942	A	0.399	2863	-0.16	-0.026	8.34 × 10^−10^	0.41	0.9997
Patients	chr9:135133193	rs545971	9	133267960	A	0.381	2864	-0.16	-0.026	5.95 × 10^−10^	0.48	1
Patients	chr9:135133263	rs612169	9	133268030	G	0.381	2864	-0.16	-0.026	5.95 × 10^−10^	0.48	1
Patients	chr9:135135292	rs582118	9	133270061	G	0.381	2864	-0.16	-0.026	5.95 × 10^−10^	0.48	1
Patients	chr9:135135305	rs582094	9	133270074	A	0.381	2864	-0.16	-0.026	5.95 × 10^−10^	0.48	1
Patients	chr9:135135795	rs2769071	9	133270565	G	0.381	2864	-0.16	-0.026	5.95 × 10^−10^	0.48	1
Patients	chr9:135136048	rs676457	9	133270797	A	0.382	2864	-0.16	-0.026	4.44 × 10^−10^	0.58	1
Patients	chr9:135137116	rs8176649	9	133271880	A	0.375	2864	0.14	0.026	1.63 × 10^−7^	0.75	1
Patients	chr9:135138917	rs8176646	9	NA	C	0.398	2864	-0.16	-0.026	8.79 × 10^−10^	0.44	1
Patients	chr9:135138919	rs8176645	9	133273682	A	0.398	2860	-0.16	-0.026	1.43 × 10^−9^	0.39	0.9986
Patients	rs505922	rs505922	9	133273813	G	0.380	2863	-0.16	-0.026	7.57 × 10^−10^	0.50	0.9997
Patients	chr9:135139321	rs529565	9	133274084	G	0.380	2864	-0.16	-0.026	9.13 × 10^−10^	0.50	1
Controls	rs11961593	rs11961593	6	165750649	A	0.067	408	0.64	0.12	1.80 × 10^−7^	0.41	1

All genome wide significant results from GWAS in ACS patients and controls separately. The table includes basic information on the original SNP name on the CardioMetaboChip, The RS number (SNP name), chromosome and position of the SNP. In addition, the table shows which allele the effect (Beta) was estimated for, the frequency of that allele and the effect (Beta) the allele have on IL-10 levels with corresponding standard error (SE) and P-value. The table also includes basic QC values for the SNPs including number of individuals successfully genotyped, SNP call rate, and Hardy-Weinberg equilibrium P-value (HWE *P*).

**Table 5:** Odds ratio for disease outcome in ACS patients with different IL-10 levels or ABO alleles.

Marker	Myocardial infarction after 5 years		Cardiovascular death after 5 years		Any cause of death after 9 years		Any cause of death or myocardial infarction after 5 years	
	**OR** ^a^ **[95% CI** ^b^ **]**	***P***	**OR [95% CI]**	***P***	**OR [95% CI]**	***P***	**OR [95% CI]**	***P***
IL-10 (1 SD ^c^ increase)	1.16 [1.04–1.29]	0.0081	1.13 [0.97–1.32]	0.13	1.26 [1.13–1.39]	1.4 × 10^−05^	1.23 [1.11–1.35]	4.9 × 10^−05^
rs676457^d^ (major allele)	1.26 [1.08–1.47]	0.0028	1.28 [1.02–1.59]	0.029	1.29 [1.11–1.5]	0.00067	1.24 [1.08–1.42]	0.0025

The table shows the odds ratio that is associated with a 1 standard deviation increase in IL-10 levels and the odds ration that is associated with having one extra copy of the major allele of rs676457. The odds ratios, the 95% confidence intervals, and corresponding P-values was estimated for three different endpoints after five our nine years in the ACS cohort.

^a^ OR–odds ratio.

^b^ CI–confidence interval.

^c^ SD–standard deviation.

^d^ The major allele in rs676457 tags the O antigen and is associated with increased IL-10 levels.

### Haplotype analyses, ABO antigens and blood groups

There are several different alleles within the coding region of the *ABO* gene resulting in four main histo-blood group antigens: O, A, B and AB [22]. The CardioMetaboChip does not include all the sequence variants that specify the different ABO antigens. However, using data from both the Seattle SNP Variation Discovery Resource (http://pga.gs.washington.edu/) and the Blood Group Antigen Mutation Database (http://www.ncbi.nlm.nih.gov), three of the SNPs on the CardioMetaboChip: rs8176746, rs687289 and rs507666 were shown in a previous study [23] to tag the main ABO antigens (Table 6). One of these, rs687289, is in LD (R = 0.996) with rs676457, our top IL-10 associated SNP. Consequently, the association between rs676457 and IL-10 in the ACS cohort represents an association between the O antigen and increased IL-10 levels, and thus the O antigen with poor disease outcome (Table 5).

**Table 6:** Frequencies of haplotypes representing the different ABO antigen alleles in ACS patients and controls.

ABO allele	SNPs			Frequency	
	rs8176746	rs687289	rs507666	Patients	Controls
A1	C	A	A	0.223	0.223
A2	C	A	G	0.084	0.091
B	A	A	G	0.074	0.083
O	C	G	G	0.618	0.601

The table shows the combination of alleles at three different SNPs that represent the four major ABO antigens. The frequency for inferred antigen is shown for ACS patients and controls separately. No difference in frequency were seen between ACS patients and controls for any of the antigens (*P*>0.05).

The three-marker haplotypes (rs8176746, rs687289 and rs507666) that tag the main ABO antigens (A1, A2, B and O) were used for haplotypes analyses (Table 6). No evidence was found that the ABO antigens influence disease status (ACS patients compared to controls, global *P* = 0.41), and the frequencies of the haplotypes did not differ between the groups (Table 6). The global score statistics using an additive model was applied to evaluate the effect of ABO antigens on a wide range of biomarkers involved in inflammation and coagulation (Table 7). After adjusting for the total number of traits tested, in addition to IL-10 both VWF and sTF were also significantly associated with the ABO haplotypes. However, unlike IL-10, both VWF and sTF were significant in both the ACS patients and controls. The association with VWF appears to be similar between patients and controls, while the association with sTF is much weaker in the patients.

**Table 7:** Haplotype score statistics for the ABO antigens and all biomarkers using an additive model.

	ACS patients	Controls
	*P*	*P*
IL-10	1.7 × 10^−8^	0.70
VWF	9.2 × 10^−13^	1.2 × 10^−15^
CD40 ligand	0.53	0.18
IL-6	0.06	0.40
Fibrinogen	0.46	0.39
IL-18	0.36	0.85
sTF	0.00086	1.03 × 10^−05^

The table shows the association between variation in the level of the biomarkers and different ABO antigens in ACS patients and controls separately.

The relative effect of individual ABO antigens on VWF, IL-10 and sTF were investigated in the ACS patients and controls separately (S1 Table). For VWF the results are very similar between the patients and controls. Antigens B and A1 correlate with significantly higher levels of VWF compared to A2 and O. Although, comparing B with A1, or O with A2, shows no difference. On the contrary, when analyzing the effect of IL-10 levels in combination with the haplotypes, the ABO antigens do have a detectable effect in the ACS patients but not in the controls which is in agreement with our previous analyses. The observed effects the individual antigens have on IL-10 levels are: higher levels for O, closely followed by A2, and much lower levels for A1 and B. The effect of the ABO antigens on the levels of sTF appears to be stronger in the controls than in the patients. While the B antigen is associated with increased sTF levels in both the patients and in the controls, the A2 allele appears to further decrease the sTF levels compared to the O antigen in the patients but not in the controls (S1 Table).

In transfusion medicine, individuals are classified according to their ABO blood group (O, A, B or AB) where: O represents homozygous O/O individuals, A represents A/A, and A/0, B represents B/B and B/0, and AB represents A/B antigen. The frequency of the different antigen combinations within the genotypes did not differ between the ACS patients and controls (*P*>0.05) (S2 Table). For VWF, there was no difference between: O/O, A2/A2 and A2/0 individuals (adjusting for patient/control status), neither between: B/B and B/0 blood group B individuals, nor between: A1/A1 and A1/0 blood group A1 individuals (*P*>0.05). Similarly, for IL-10, no difference was seen between either homozygous A1/A1 or B/B patients, compared to the heterozygous A1/O or B/O patients. These results suggest that the A1 and B antigens are dominant over O with regards to IL-10 and VWF levels. However, the small sample sizes, makes detecting a statistically significant difference between the different groups difficult. When it comes to sTF, both homozygous B and B/0 heterozygotes (blood group B) have higher levels of sTF compared to the other blood groups, but there was no difference between the heterozygous B/0 and homozygous B/B blood group B individuals (*P*>0.05). On the other hand, the A2 antigen was associated with lower sTF levels, and this effect was even stronger in A2/A2 homozygotes (S2 Table) (*P* = 0.050).

### Path analyses

**Fig 2:**
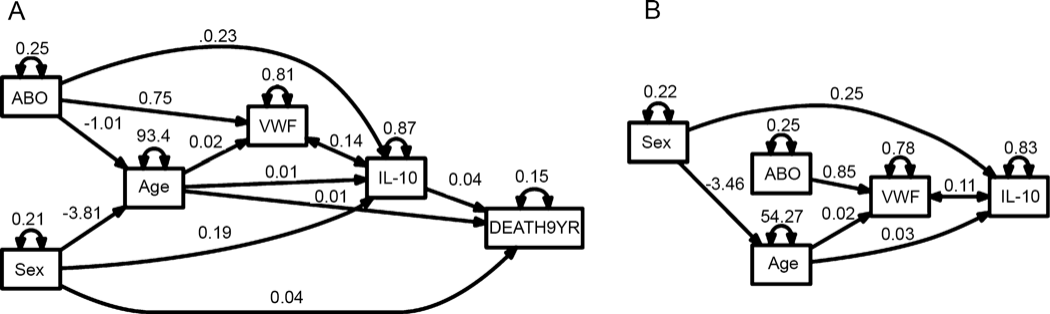
Path analyses in ACS patients (A) and controls (B). Single-headed arrows are direct associations represented by the regression coefficient from multiple regressions. Double-headed arrows above the variables represent residual variance (for endogenous variables) or variance (for exogenous variables). Double-headed arrows between two variables are indirect associations represented by the correlation coefficient. VWF and IL-10 are both standardized. Only significant (*P*<0.05) associations are shown in the figure. Note that age, as a responding variable in the ACS patients, should be regarded as the age of onset.

Path analyses (Fig 2) were performed in patients (final model: *P* = 0.15, Adjusted goodness-of-fit index = 0.996, RMSEA index = 0.0154) and controls (final model: *P* = 0.73, Adjusted goodness-of-fit index = 0.993, RMSEA index<0.0001) separately. Although the regression and correlation coefficients are similar for most variables, the effect the ABO antigens have on age and IL-10 levels are only observed in the patient group (Fig 2, Table 8). We also identified a difference in the age of onset between the sexes, where females were older at onset of ACS than men. This association is also seen in the controls, since the controls are matched pair-wisely for sex and age with a subset of the ACS patients. It is also worth noting that while the O and A2 blood group have increased IL-10 levels, but decreased VWF, there is still a positive correlation between IL-10 and VWF in both the patients and the controls.

**Table 8:** Coefficients and direction of effects from the path analyses.

	Variable 1	Direction	Variable 2	Patients		Controls	
Coefficients from path analyses				Estimate	*P*	Estimate	*P*
Regression	IL-10	<—-	ABO (A, B, AB)	-0.23	2.2 × 10^−11^	0.068	4.5 × 10^−1^
Regression	VWF	<—-	ABO (A, B, AB)	0.75	8.0 × 10^−111^	0.85	4.2 × 10^−23^
Regression	Death^a^	<—-	IL-10	0.038	4.9 × 10^−7^	NA	NA
Regression	Age	<—-	ABO (A, B, AB)	-1.01	4.6 × 10^−3^	0.55	4.5 × 10^−1^
Regression	Age	<—-	Sex (Males)	-3.81	2.6 × 10^−22^	-3.46 ^b^	6.5 × 10^−6^
Regression	IL-10	<—-	Age	0.012	1.7 × 10^−11^	0.031	3.9 × 10^−7^
Regression	VWF	<—-	Age	0.025	3.4 × 10^−48^	0.024	2.5 × 10^−5^
Regression	IL-10	<—-	Sex	0.19	7.5 × 10^−7^	0.253	8.7 × 10^−3^
Regression	Death^a^	<—-	Age	0.013	4.1 × 10^−75^	NA	NA
Regression	Death^a^	<—-	Sex (Males)	0.037	2.1 × 10^−2^	NA	NA
Correlation	IL-10	<—>	VWF	0.14	6.7 × 10^−19^	0.11	4.9 × 10^−3^

The table shows the main results from the path analyses with regression or correlation coefficients (estimates) for ACS patient and controls separately and respective P-value. Only variables with a P-value < 0.05 are included.

^a^ Death is the all-cause mortality after 9 years.

^b^ Controls were pair wisely matched (regarding sex and age) with a subset of the ACS patients.

### Interaction with time of blood sampling

A possible confounder in the analyses is the time delay between the onset of the last chest pain to the time of blood sampling. Though, we found no evidence that this time delay influenced neither the IL-10 levels (*P =* 0.85), the association between the ABO blood groups, nor the variation in IL-10 levels (*P =* 0 .65). However, VWF levels did increase as the sampling time was delayed (*P =* 3.22 × 10^−6^).

## Discussion

We have shown that the ABO antigens play important roles for the inflammatory response in ACS patients. More specifically, they are genetic determinants of IL-10 levels in these patients. The time delay from the last event of chest pain to the time of blood sampling does not appear to influence the levels of IL-10, and consequently we can assume that the observed increase in IL-10 levels is due to an overall increased inflammatory activity in the arteries in these patients. This is in agreement with IL-10 being associated with an increased risk for all the clinical endpoints investigated in this study.

### ABO blood group system

It is well known that ABO blood group typing is of great importance when receiving blood transfusions. The ABO gene consists of seven exons and has three main alleles: A, B and O. The ABO gene encodes the H-antigen, which is the precursor of the A, B and O-antigens. Depending on an individual’s ABO genotype, the protein will contain several different amino acid substitutions creating the specificity for the different antigens. The A and B allele encodes their own specific A- and B- glycosyltransferase that binds to the H-antigen and modifies it. The A and B antigens are produced and expressed predominantly in red blood cells (RBC), resulting in A or B-antigens being expressed on the surface of RBCs. The O allele of the ABO gene is created by a single nucleotide change in exon 6. This causes a frame shift resulting in the loss of the transferase activity leaving the H-antigen “uncharged”, resulting in neither A nor B-antigens being expressed on the RBCs [22]. Individuals of blood group A, B or AB also have some “uncharged” H-antigens, but at a lower level compared to the O blood group. The main ABO antigens can further be divided into a large number of subgroups [24]. The most functionally relevant subgroup is the subdivision of the A-antigen into variants A1 and A2, where A1 has 10–20 times higher enzyme activity compared to the A2 variant [25].

### ABO antigens and biomarkers

Even though ABO antigens are mainly expressed on the surface of RBCs, ABO antigens are also present on most epithelial and endothelial cells, on T-cells, B-cells and platelets as well as detectable in most body fluids such as saliva [26]. Moreover, the ABO antigens are also expressed on VWF [27,28], and the interaction between the ABO antigens and VWF is complex. Individuals that are homozygous for the O allele have previously been shown to have dramatically lower levels of VWF (and Factor VIII) compared to individuals with either A or B antigens [29,30]. One explanation might be that the binding of A, B or H-antigens affects the clearance rate of VWF from the blood stream. VWF is a coagulation factor and not surprisingly, individuals that are homozygous for the O allele are predisposed to bleeding [31]. Interestingly, in our data shows that the ABO antigens also influence the level of sTF, another important component of the coagulation cascade, although here the effect of the different ABO antigens on sTF levels appears to be even more complex. In our study, we replicated previous findings of the association between the ABO O-antigen with lower levels of VWF [29,30]. Additionally, we also find that the ABO A2 antigen shows similar effects on both VWF and IL-10 levels as the O antigen. The absence of active antigens in homozygous O individuals and a much lower activity of A2 compared to A1 and B, suggests that low enzyme activity is the main determinant of both the increase in IL-10 levels and the decrease in VWF levels.

### ABO blood groups and disorders

In several previous studies the ABO O blood group has been associated with decreased blood coagulation and a reduced risk of venous thromboembolism [32] as well as with CAD [17]. Analyses using our data do not detect a difference in the frequency of neither the ABO antigens, nor the blood groups between the ACS patients and the controls. However, we do observe an association between the ABO antigens and the clinical endpoint in the ACS patients, but the higher age of onset in ACS individuals with blood groups O and A2 might overestimate the poor disease progress. The increased age of onset in the individuals with O or A2 blood groups could be due to lower coagulation properties, e.g. mediated by lower levels of VWF. Still, inflammation is another major contributor to CAD [17], making it reasonable to assume that inflammation is a much more pronounced ACS risk factor in individuals of blood group O or A2 compared to A1 and B, as reflected by the increase in IL-10 levels in these individuals. The ABO antigens have previously been associated with a variation in the inflammatory response, especially with levels of IL-6, another inflammatory marker [33]. O blood group individuals also show an increased inflammatory response to *Helicobacter pylori* infections, but there it was associated with an increase in IL-6, but not IL-10 secretion [34].

### Limitations

There are some limitations with this study. The GWAS was performed using the CardioMetaboChip, which is specifically enriched for SNPs and gene targets from previously conducted studies on cardiovascular and metabolic diseases. This chip included a large number of SNPs in the ABO region. However, very few SNPs in the *IL10* (the gene coding for IL-10) region are present on this chip and we can therefore not exclude that e.g. promoter SNPs in the *IL10* might also influence the levels of IL-10.

Our data showed that the O antigen was associated with poor disease outcome, and using path analyses we identified a negative effect of the A and B antigens on age in ACS patients. This probably reflects the higher age of onset of ACS in individuals with O and A2 blood groups, and consequently, the effect of the ABO antigens on clinical endpoints might be partly mediated by an increased age of onset.

Previous studies have also shown that another blood group system, Rhesus (Rh) factor (D antigen), influence the mortality rate in patients with ACS when comparing Rh-positive vs. Rh-negative blood groups [35]. In our study we have only focused on the ABO blood group system and not on Rh.

It is also worth pointing out that all biomarkers were not measured in the complete dataset. While IL-10 was measured in all patients and controls, sTF and VWF was only measured in a subset of the patents (N<800) and the power to identify ABO associations with some of the biomarkers might therefore be limited.

### Clinical perspective

ACS is a heterogeneous disease with many contributing risk factors, and the effect of many genetic variants (including the ones determining the ABO antigens), have been associated with the risk of developing ACS or CAD [17]. However, it is interesting that we do see a clear difference between the ABO antigens and the age of onset of ACS. Individuals with blood group O are protected against ACS due to their lower coagulation properties, but the blood group type O patients also have higher IL-10 levels. One possible explanation for this might be that the main cause of ACS in individuals of blood group A, B, or AB is increased coagulation properties, while inflammation is the main risk factor in individuals of blood group type O. So far, no study has shown any causal role of IL-10 on ACS, and we therefore suggest that increased IL-10 levels is reflecting an underlying inflammatory state, rather than being directly causal of ACS. Consequently, patients of blood group type O might be more suitable for treatment by anti-inflammatory drugs to decrease their risk of recurrent myocardial infarction, a strategy the might be worth examining in the future.

### Conclusion

We have identified that the ABO antigens 0 and A2 are associated with increased IL-10 levels and decreased VWF levels, and that the ABO antigens also influence the levels of sTF. We further also show that the ABO antigens are associated with disease outcomes in ACS patients. It is evident that several molecular processes as well as the underlying genetic content influence the development of ACS, but what these underlying causes and associations are still remains to be elucidated. Our findings do form a founding platform for further investigations into the link between the ABO antigens, biomarkers and clinical outcomes in ACS, which will be important for both individualized treatment and drug discovery in the future.

## Supporting Information

S1 Fig The first (PC1) and second (PC2) principal component for the ACS cohort. The total cohort consists of ACS patients and controls from Sweden, Denmark and Norway. In A) the colors in the plot represent the country of origin: Sweden (red), Denmark (green), or Norway (blue). In B) the colors in the plot indicate if an individual is an ACS patient (red) or a control (blue).
(DOCX)

S2 Fig Distribution of the untransformed biomarker levels. Each histogram shows the number of individuals with a certain biomarker level. All values are untransformed and no outliers have been removed.
(DOCX)

S3 Fig Q-Q plots for the IL-10 GWAS. The figures show the observed Chi-square value from the test statics plotted against the expected Chi-square value if there is no association between IL-10 and any of the genetic variants investigated in: A) for the GWAS in the ACS patients, and B) for the GWAS in the controls. The black line in the figures has the slope 1.0 and the red line is the slope of the points in the plot indicating if there is an inflation in low P-value (high Chi-square values) in the analyses. The slope of the red line is 1.038 for the GWAS in the ACS patients and 1.019 for the GWAS in the controls.
(DOCX)

S4 Fig Pair-wise LD pattern between the SNPs in the ABO genes. The figure shows the pair-wise correlation coefficient (R^2^ values) between the SNPs in the ABO gene. Red indicates high LD within a block (R^2^ > 0.47), and pink indicates intermediate LD (0.3 ≤ R^2^ ≥ 0.47). The uncolored pair-wise comparisons indicate low LD (R^2^ < 0.3). The Marker names in bold in yellow are the ones that together represent the ABO antigens: A1, A2, O and B. The Markers in bold are the ones that were significantly associated with IL-10 levels in the GWAS. Included in the figure is the original name of the SNPs used on the CardioMetaboChip, the RS number for the, the position on chromosome 9 (Build 38), and the frequency in our total cohort (ACS patients + controls). The five LD blocks labeled in the figure is defined by high LD between all SNPs in the block (R^2^ > 0.47).
(DOCX)

S1 Table Comparison of how the biomarkers are affected by the different ABO antigens in ACS patients and controls. The table shows the increase (positive) or decrease (negative) in the mean value between individuals with different ABO antigens (Antigen 2 compared to Antigen 1) and respective *P*-value for the three biomarkers investigated.
(DOCX)

S2 Table Biomarker values for: the different ABO blood type groups, and for each combination of antigens. The table shows the mean and standard deviation for the levels of the three biomarkers in individuals with different ABO antigens combinations in ACS patients and controls separately. The table also includes the number of individuals and the carrier frequency of each antigen combination in the ACS patients and controls, as well as the number of individuals where biomarkers have been measured.
(DOCX)

S3 Table GWAS results for all SNPs. The table show the GWAS results for ACS patients and controls separately as well as the results for the two cohorts pooled together.
(XLSX)
