# Supplementary material for: Genome-Wide Association Study Identifies That the ABO Blood Group System Influences Interleukin-10 Levels and the Risk of Clinical Events in Patients with Acute Coronary Syndrome
Source: PLoS One. 2015 Nov 24;10(11):e0142518. doi: 10.1371/journal.pone.0142518 (PMC4658192; doi:10.1371/journal.pone.0142518)
Supplement: S1 Fig — The total cohort consists of ACS patients and controls from Sweden, Denmark and Norway. In A) the colors in the plot represent the country of origin: Sweden (red), Denmark (green), or Norway (blue). In B) the colors in the plot indicate if an individual is an ACS patient (red) or a control (blue). (DOCX) [file pone.0142518.s001.docx]

A B


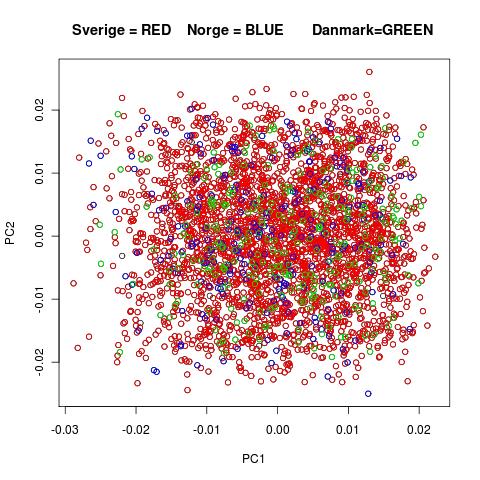

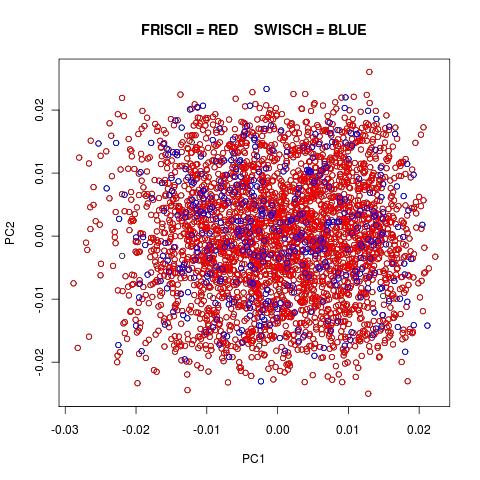


**S1 Fig. The first (PC1) and second (PC2) principal component for the ACS cohort.** The total cohort consists of ACS patients and controls from Sweden, Denmark and Norway. In A) the colors in the plot represent the country of origin: Sweden (red), Denmark (green), or Norway (blue). In B) the colors in the plot indicate if an individual is an ACS patient (red) or a control (blue).
