# Supplementary material for: Genome-Wide Association Study Identifies That the ABO Blood Group System Influences Interleukin-10 Levels and the Risk of Clinical Events in Patients with Acute Coronary Syndrome
Source: PLoS One. 2015 Nov 24;10(11):e0142518. doi: 10.1371/journal.pone.0142518 (PMC4658192; doi:10.1371/journal.pone.0142518)
Supplement: S2 Fig — Each histogram shows the number of individuals with a certain biomarker level. All values are untransformed and no outliers have been removed. (DOCX) [file pone.0142518.s002.docx]

A B C

**S2 Fig. Distribution of the untransformed biomarker levels.** Each histogram shows the number of individuals with a certain biomarker level: A) IL-10, B) sTF, and C) vWF. All values are untransformed and no outliers have been removed.
