# Supplementary material for: Genome-Wide Association Study Identifies That the ABO Blood Group System Influences Interleukin-10 Levels and the Risk of Clinical Events in Patients with Acute Coronary Syndrome
Source: PLoS One. 2015 Nov 24;10(11):e0142518. doi: 10.1371/journal.pone.0142518 (PMC4658192; doi:10.1371/journal.pone.0142518)
Supplement: S3 Fig — The figures show the observed Chi-square value from the test statics plotted against the expected Chi-square value if there is no association between IL-10 and any of the genetic variants investigated in: A) for the GWAS in the ACS patients, and B) for the GWAS in the controls. The black line in the figures has the slope 1.0 and the red line is the slope of the points in the plot indicating if there is an inflation in low P-value (high Chi-square values) in the analyses. The slope of the red line is 1.038 for the GWAS in the ACS patients and 1.019 for the GWAS in the controls. (DOCX) [file pone.0142518.s003.docx]

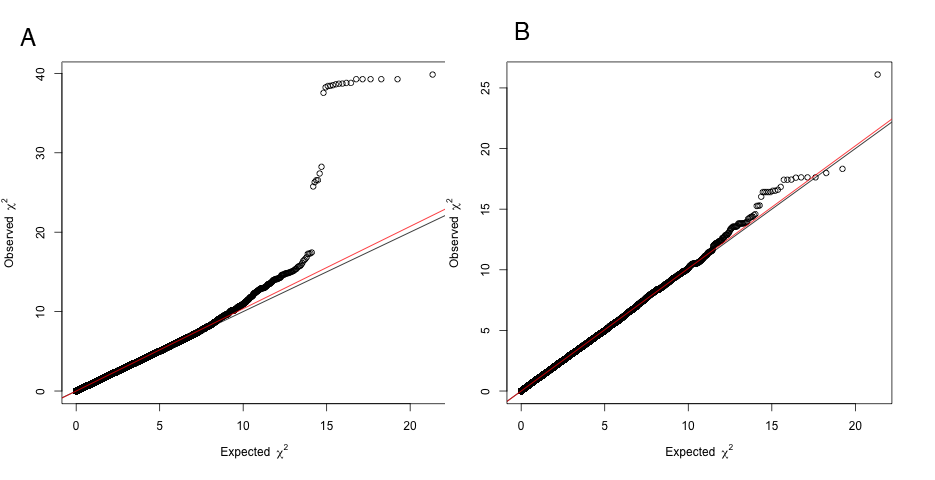


**S3 Fig. Q-Q plots for the IL-10 GWAS.** The figures show the observed Chi-square value from the test statics plotted against the expected Chi-square value if there is no association between IL-10 and any of the genetic variants investigated in A) ACS patients, and B) Controls. The black line in the figures has the slope one and the red line is the slope of the points in the plot indicating if there is an inflation in low P-value (high Chi-square values) in the analyses. The slope of the red line is 1.038 in the ACS patients and 1.019.
