# Supplementary material for: Genome-Wide Association Study Identifies That the ABO Blood Group System Influences Interleukin-10 Levels and the Risk of Clinical Events in Patients with Acute Coronary Syndrome
Source: PLoS One. 2015 Nov 24;10(11):e0142518. doi: 10.1371/journal.pone.0142518 (PMC4658192; doi:10.1371/journal.pone.0142518)
Supplement: S4 Fig — The figure shows the pair-wise correlation coefficient (R2 values) between the SNPs in the ABO gene. Red indicates high LD within a block (R2 > 0.47), and pink indicates intermediate LD (0.3 ≤ R2 ≥ 0.47). The uncolored pair-wise comparisons indicate low LD (R2 < 0.3). The Marker names in bold in yellow are the ones that together represent the ABO antigens: A1, A2, O and B. The Markers in bold are the ones that were significantly associated with IL-10 levels in the GWAS. Included in the figure is the original name of the SNPs used on the CardioMetaboChip, the RS number for the, the position on chromosome 9 (Build 38), and the frequency in our total cohort (ACS patients + controls). The five LD blocks labeled in the figure is defined by high LD between all SNPs in the block (R2 > 0.47). (DOCX) [file pone.0142518.s004.docx]

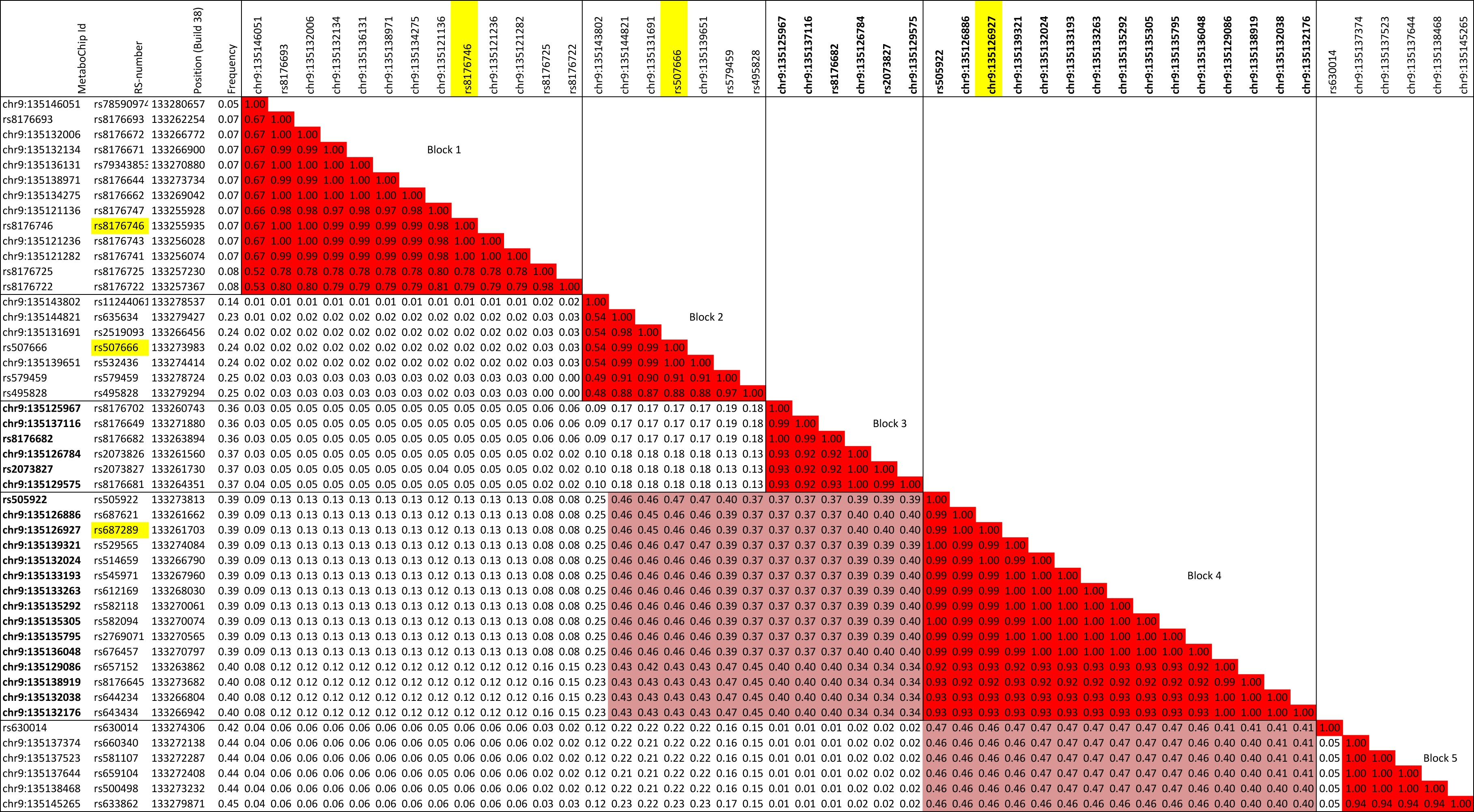
­­

**S4 Fig. Pair-wise LD pattern between the SNPs in the ABO genes.** The figure shows the pair-wise correlation coefficient (R^2^ values) between the SNPs in the ABO genes. Red indicates high LD within a block (R^2^ > 0.47), and pink indicates intermediate LD (0.3 ≤ R^2^ ≥ 0.47). The uncolored pair-wise comparisons indicate low LD (R^2^ < 0.3). The Marker names in bold in yellow are the ones that together represent the ABO antigens: A1, A2, O and B. The Markers in bold are the ones that were significantly associated with IL-10 levels in the GWAS. Included in the figure is the original name of the SNPs used on the CardioMetaboChip, the RS number for the, the position on chromosome 9 (Build 38), and the frequency in our total cohort (ACS patients + controls). The five LD blocks labeled in the figure is defined by high LD between all SNPs in the block (R^2^ > 0.47).
