# Supplementary material for: Genome-Wide Association Study Identifies That the ABO Blood Group System Influences Interleukin-10 Levels and the Risk of Clinical Events in Patients with Acute Coronary Syndrome
Source: PLoS One. 2015 Nov 24;10(11):e0142518. doi: 10.1371/journal.pone.0142518 (PMC4658192; doi:10.1371/journal.pone.0142518)
Supplement: S1 Table — The table shows the increase (positive) or decrease (negative) in the mean value between individuals with different ABO antigens (Antigen 2 compared to Antigen 1) and respective P-value for the three biomarkers investigated. (DOCX) [file pone.0142518.s005.docx]

**S1 Table. Comparison of how the biomarkers are affected by the different ABO antigens in ACS patients and controls.**

| **Biomarker** | **Antigen 1** | **Antigen 2** | **Antigen 2 compared to antigen 1** | | | |
| --- | --- | --- | --- | --- | --- | --- |
|  |  |  | **Controls** | | **Patients** | |
|  |  |  | **Beta^a^** | ***P*** | **Beta^a^** | ***P*** |
| sTF | O | A2 | -3.53 | 0.676 | -22.9 | 0.0064 |
| sTF | O | A1 | -14.69 | 0.0151 | -11.2 | 0.03 |
| sTF | O | B | 42.86 | 5.9*10^-6^ | 9.69 | 0.25 |
| sTF | A2 | A1 | -12.091 | 0.155 | 11.6 | 0.19 |
| sTF | A2 | B | 45.349 | 8.9*10^-5^ | 32.4 | 0.0031 |
| sTF | A1 | B | 56.926 | 5.8*10^-8^ | 20.8 | 0.020 |
| IL-10 | O | A2 | -0.05 | 0.652 | -0.0979 | 0.034 |
| IL-10 | O | A1 | 0.0291 | 0.719 | -0.1586 | 1.7*10^-7^ |
| IL-10 | O | B | 0.0718 | 0.562 | -0.2299 | 1.6*10^-6^ |
| IL-10 | A2 | A1 | 0.0632 | 0.597 | -0.0635 | 0.21 |
| IL-10 | A2 | B | 0.1043 | 0.51 | -0.1349 | 0.029 |
| IL-10 | A1 | B | 0.0355 | 0.798 | -0.0726 | 0.16 |
| VWF | O | A2 | -0.118 | 0.975 | -0.13 | 0.98 |
| VWF | O | A1 | 21.525 | 1.6*10^-14^ | 20.6 | 6.4*10^-11^ |
| VWF | O | B | 21.934 | 1.9*10^-7^ | 30.6 | 2.2*10^-9^ |
| VWF | A2 | A1 | 20.52 | 6.1*10^-8^ | 21.1 | 6.4*10^-7^ |
| VWF | A2 | B | 20.81 | 0.0000434 | 31.2 | 3.0*10^-7^ |
| VWF | A1 | B | 0.104 | 0.982 | 10.3 | 0.059 |

The table shows the increase (positive) or decease (negative) in mean value between individuals with different ABO antigens (Antigen 2 compared to Antigen 1) and respective P-value for the three biomarkers investigated.

^a^ Beta – The increase in biomarker for Antigen 2 compared to Antigen 1
