# Supplementary material for: Genome-Wide Association Study Identifies That the ABO Blood Group System Influences Interleukin-10 Levels and the Risk of Clinical Events in Patients with Acute Coronary Syndrome
Source: PLoS One. 2015 Nov 24;10(11):e0142518. doi: 10.1371/journal.pone.0142518 (PMC4658192; doi:10.1371/journal.pone.0142518)
Supplement: S2 Table — The table shows the mean and standard deviation for the levels of the three biomarkers in individuals with different ABO antigens combinations in ACS patients and controls separately. The table also includes the number of individuals and the carrier frequency of each antigen combination in the ACS patients and controls, as well as the number of individuals where biomarkers have been measured. (DOCX) [file pone.0142518.s006.docx]

**S2 Table. Biomarker values for: the different ABO blood type groups, and for each combination of antigens**

| **Antigens** | **Blood group** | **Controls** | | **Patients** | | **VWF** | | | | **IL-10** | | | | **sTF** | | | |
| --- | --- | --- | --- | --- | --- | --- | --- | --- | --- | --- | --- | --- | --- | --- | --- | --- | --- |
|  |  |  |  |  |  | **Controls** | | **Patients** | | **Controls** | | **Patients** | | **Controls** | | **Patients** | |
|  |  | **N** | **Freq** | **N** | **Freq** | **N** | **Mean**  **(SD)** | **N** | **Mean**  **(SD)** | **N** | **Mean**  **(SD)** | **N** | **Mean**  **(SD)** | **N** | **Mean**  **(SE)** | **N** | **Mean**  **(SE)** |
| **A1A1** | **A1** | 19 | 0.046 | 163 | 0.056 | 19 | 162  (29) | 33 | 167  (40) | 19 | -0.239  (0.644) | 158 | -0.05  (0.944) | 19 | 132  (67) | 52 | 182  (100) |
| **A1A2** | **A1A2** | 10 | 0.024 | 101 | 0.035 | 10 | 155  (42) | 14 | 161  (36) | 10 | -0.399  (0.555) | 101 | -0.004  (1.083) | 10 | 140(  92) | 27 | 139  (75) |
| **A1B** | **A1B** | 21 | 0.051 | 88 | 0.03 | 20 | 151  (27) | 14 | 168  (33) | 20 | -0.293  (0.492) | 84 | -0.017  (1.038) | 20 | 126  (35) | 27 | 166  (88) |
| **A2A2** | **A2** | 6 | 0.015 | 16 | 0.005 | 6 | 110  (21) | 2 | 99  (1) | 6 | -0.218  (1.039) | 16 | 0.247  (0.884) | 5 | 148  (92) | 3 | 77  (24) |
| **A2B** | **A2B** | 9 | 0.022 | 28 | 0.01 | 9 | 148  (17) | 9 | 160  (33) | 9 | -0.343  (0.585) | 28 | 0.067  (1.045) | 9 | 179  (73) | 6 | 201  (71) |
| **BB** | **B** | 0 | 0 | 21 | 0.007 | 0 | NA | 5 | 187  (26) | 0 | NA | 20 | -0.214  (0.816) | 0 | NA | 5 | 202  (103) |
| **OA1** | **A1** | 115 | 0.279 | 789 | 0.27 | 115 | 143  (33) | 142 | 166  (40) | 114 | -0.296  (0.681) | 778 | -0.052  (1.021) | 113 | 124  (66) | 211 | 168  (84) |
| **OA2** | **A2** | 44 | 0.107 | 328 | 0.112 | 44 | 116  (31) | 54 | 144  (50) | 44 | -0.392  (0.568) | 318 | 0.034  (1.014) | 42 | 126  (62) | 82 | 171  (73) |
| **OB** | **B** | 38 | 0.092 | 277 | 0.095 | 38 | 147  (28) | 39 | 188  (53) | 38 | -0.267  (0.581) | 272 | -0.125  (0.918) | 36 | 206  (96) | 72 | 200  (92) |
| **OO** | **O** | 148 | 0.359 | 1106 | 0.379 | 146 | 118  (31) | 189 | 136  (41) | 146 | -0.346  (0.553) | 1085 | 0.24  (0.948) | 146 | 138(63) | 271 | 188  (85) |

The table shows the mean and standard deviation in levels of three biomarkers in individuals with different ABO antigens combinations in ACS patients and controls separately. The table also includes the number of individuals and the carrier frequency of each antigen combination in the ACS patients and controls, as well as the number of individuals where biomarkers have been measured.
